# Fasting Blood Glucose and Depressive Mood among Patients with Mental Illness in a Medicaid Managed Care Program

**DOI:** 10.1155/2011/862708

**Published:** 2011-05-26

**Authors:** Linda S. Kahn, Roger S. McIntyre, Lisa Rafalson, Diane E. Berdine, Chester H. Fox

**Affiliations:** ^1^Department of Family Medicine, School of Medicine and Biomedical Sciences, University at Buffalo, The State University of New York, NY 14215, USA; ^2^Mood Disorders Psychopharmacology Unit, University Health Network, Toronto, ON, Canada M5T 2S8; ^3^Department of Psychiatry, University of Toronto, Toronto, ON, Canada M5T 2S8; ^4^Gold Choice, UB Family Medicine, Inc., Buffalo, NY 14215, USA

## Abstract

*Objective*. This study explores the relationship between depressive symptoms, as measured by the PHQ-9 depression screen and blood glucose levels among patients with diabetes enrolled in Gold Choice, a Medicaid managed care program for individuals with mental illness and/or substance abuse. *Methods*. The PHQ-9 was mailed to 454 Gold Choice members and a questionnaire was mailed to their physicians requesting current HbA1c% and fasting blood glucose (FBG) levels. The pearson product-moment correlation was used to describe the association between PHQ-9 scores and FBG levels. *Results*. The PHQ-9 response rate was 55% (*N* = 249). Laboratory results were received for 141 patients. The correlation between FBG and PHQ-9 scores was modest but statistically significant: *r* = 0.21 , *P* = 0.015. *Conclusion*. A statistically significant association was found between FBG and PHQ-9 depression scores. This finding supports current recommendations that physicians be alert to depressive symptoms among patients with diabetes or impaired glucose metabolism.

## 1. Introduction

The co-occurrence of mood disorders and diabetes is well documented, and both conditions frequently present together in the primary care setting [1]. Patients with diabetes and mood disorders exhibit a more severe course of diabetes, with greater morbidity and higher mortality, compared to those without mood disorders [2–4]. Broadly sketched, a similar composite emerges for both conditions, high prevalence in the general population, low case detection, protracted illness course high rates of comorbidity, multifactorial etiology, substantial illness burden and economic cost, and association with preventable and premature excess mortality [5, 6]. 

Both depression and diabetes are associated with altered metabolic pathways and stress response networks, resulting in disruptions in glucose transport and insulin resistance [7–9]. It remains unknown, however, if the dimensional severity of depressive symptoms corresponds with the progressive disruption of glucose homeostasis. Studies that have explored the relationship between glycemic control and depressive symptoms have yielded mixed results and the direction of the association remains unclear [2, 10–16]. 

In this paper, we explored the relationship between depressive symptoms, as measured by the 9-item depression scale of the Patient Health Questionnaire (PHQ-9) and blood glucose levels among patients with diabetes enrolled in Gold Choice. Gold Choice is a partially capitated Medicaid managed care program in Western New York for individuals with serious mental illness and/or substance abuse [17]. To our knowledge, no existing investigation has primarily evaluated this relationship in the patient population we have studied, a subpopulation with a higher risk for disparate chronic medical disorders.

## 2. Materials and Methods

As part of a quality assurance initiative, the PHQ-9 depression screen was mailed to 454 Gold Choice members diagnosed with diabetes, as defined by primary care encounter data. A total of 249 PHQ-9 screens were completed and returned to Gold Choice. Characteristics of this initial sample have been described elsewhere [17]. 

Physician Self-Assessment (PSA) surveys were sent to the primary care providers of the 249 patients that completed the PHQ-9 requesting the last-known HbA1c% and fasting blood glucose levels (laboratory data). The PSA is a brief self-report questionnaire that Gold Choice uses as an alternative to full-scale chart audits as well as to assess guideline adherence for standards of care as outlined in the Gold Choice Quality Assurance Plan [18]. 

Data analyses were conducted using SPSS 16.0 (Chicago, Ill, USA). Chi-square tests, *t*-tests, analysis of variance (ANOVA), and Pearson's correlation were used as appropriate. A one-sample Kolmogorov-Smirnov test confirmed normality of the distribution of PHQ-9 scores. All tests were two sided, and a *P* value <0.05 was considered statistically significant. This project was approved by the University of Toronto Institutional Review Board.

## 3. Results

In the initial study, there were no statistically significant differences observed between the 249 Gold Choice members completing the PHQ-9 and the 205 who did not return a completed screen with respect to gender or age [17]. A total of 163/249 (65%) PSA surveys were received from the primary care providers. Fasting blood glucose (FBG) and/or HbA1c laboratory results were available on 141 patients of the 163 PSA surveys that were returned to Gold Choice. 

Demographic data and depression status were compared for the patients for whom laboratory results were available and those for whom results were not available.  Table 1 shows the demographic characteristics and depression status of the sample. No statistically significant differences were observed between the two groups with respect to gender, age, or ethnicity. 

There were no statistically significant differences in depression status, as measured by the PHQ-9 score, and depression diagnosis between the group for whom laboratory results were received compared to those for whom laboratory results were not available.

Because the chi-square results for ethnicity and PHQ-9 severity level between the two groups approached significance (*P* = 0.06), we undertook additional chi-square tests to explore a possible association between ethnicity and depression severity, as measured by PHQ-9 score. We observed a statistically significant association between ethnicity and depression severity (*P* = 0.011), with Hispanic patients scoring higher than the other three groups in the moderately severe depression and severe Depression categories (data not shown). Furthermore, mean FBG concentrations did not significantly vary by ethnic group (*P* = 0.697)

Over half of the participants (55%) in the total sample of 249 screened positive for moderate depression (PHQ-9 ≥ 10). The mean HbA1c % was 7.27 (±1.7), and the mean FBG was 138 mg/dL (±68). While the correlation between HbA1c and PHQ-9 scores was not statistically significant, there was a significant association between FBG and the PHQ-9 scores: *r* = 0.21,   *P* = 0.015. There was a moderately strong correlation between HbA1c and FBG: *r* = 0.53, *P* < 0.001.

## 4. Conclusions

We observed a positive correlation between elevated fasting blood glucose and elevated PHQ-9 depression scores among patients diagnosed with diabetes. Moreover, we found few differences between patients for whom laboratory values were available and those for whom laboratory values were not available. We had anticipated that the latter group might have had worse depression, reflected in a lower receipt of laboratory tests. Our results suggest that Hispanic patients in the sample may have had more severe depressive symptoms than the other groups. The focus of a separate analysis may attempt to parse factors that portend a greater risk in Hispanic individuals. 

The present study extends and expands the growing literature documenting the relationship between glucose metabolism and depressive symptoms [10, 11, 14, 16, 20]. The cross-sectional design of this study cannot show cause/effect between elevated fasting blood glucose and depressive symptoms. It may be notable, however, that several previous studies have suggested that depressed patients experience improved glucose metabolism after treatment with antidepressants as well as cognitive behavioral therapy [21–23]. 

To our knowledge, this is the first study to evaluate the relationship between fasting blood glucose and depressive symptoms in a Medicaid managed care population of individuals with serious mental illness and/or substance abuse. Study limitations include a relatively small sample. Depression assessment was based on self-report (PHQ-9) rather than a diagnostic psychiatric interview (e.g., SCID). Because Gold Choice is a partially capitated managed-care organization that is reimbursed by Medicaid for patients' primary care services only, it does not have access to specialty or behavioral healthcare data, including psychiatric diagnoses, unless the member shares this information with his/her primary care physician, which is then transmitted to Gold Choice via primary care encounter data. These limitations reflect the methodological challenges inherent in collecting and assessing complex patient data in a Medicaid population, as well as eliciting patient information from physicians.

These findings need to be replicated in a larger sample in the general population of individuals without an existing diabetes diagnosis. Nonetheless, our preliminary finding lends further credence to current recommendations that physicians be alert to the presence of depressive symptoms among patients with diabetes or impaired glucose metabolism. 

## Figures and Tables

**Table 1:** Characteristics of the sample.

Variable	Lab data available *N* = 141	No lab data *N* = 108	*P* value
**Demographics**			

	***N*** (%)*		
**Gender**			0.41
Male	48 (35%)	42 (40%)	
Female	91 (66%)	64 (60%)	

**Age**			0.41
18–30	1 (1%)	0 (0%)	
31–40	14 (10%)	17 (16%)	
41–50	42 (30%)	32 (31%)	
50+	82 (59%)	56 (53%)	

**Ethnicity**			0.06
Hispanic	27 (19%)	28 (27%)	
African-American	56 (40%)	51 (49%)	
Caucasian	49 (35%)	23 (22%)	
Other	7 (5%)	2 (2%)	

**Depression status**			

	**Mean (sd)**		
**PHQ-9 score **	10.9 (7.4)	11.1 (6.7)	0.90
	***N*** (%)*		
**PHQ-9 Score **			0.69
≥10 (depressed)	80 (57%)	58 (54%)	
<10 (not depressed)	61 (43%)	49 (46%)	
**PHQ-9 severity level** [19]			0.06
Minimal (0–4)	36 (26%)	20 (19%)	
Mild (5–9)	25 (18%)	29 (27%)	
Moderate (10–14)	26 (18%)	29 (27%)	
Moderately severe (15–19)	34 (24%)	15 (14%)	
Severe (20–27)	20 (14%)	14 (13%)	
Depression diagnosis**	65 (61%)	42 (39% )	0.28

*Some *N*s will vary due to missing data.

**Based on primary care encounter data.

## References

[1] Anderson RJ, Freedland KE, Clouse RE, Lustman PJ (2001). The prevalence of comorbid depression in adults with diabetes: a meta-analysis. *Diabetes Care*.

[2] Lustman PJ, Anderson RJ, Freedland KE, de Groot M, Carney RM, Clouse RE (2000). Depression and poor glycemic control: a meta-analytic review of the literature. *Diabetes Care*.

[3] Katon WJ, Rutter C, Simon G (2005). The association of comorbid depression with mortality in patients with type 2 diabetes. *Diabetes Care*.

[4] de Groot M, Anderson R, Freedland KE, Clouse RE, Lustman PJ (2001). Association of depression and diabetes complications: a meta-analysis. *Psychosomatic Medicine*.

[5] Kessler RC, McGonagle KA, Zhao S (1994). Lifetime and 12-month prevalence of DSM-III-R psychiatric disorders in the United States. Results from the National Comorbidity Survey. *Archives of General Psychiatry*.

[6] American Diabetes Association (2008). Diagnosis and classification of diabetes mellitus. *Diabetes Care*.

[7] Musselman DL, Betan E, Larsen H, Phillips LS (2003). Relationship of depression to diabetes types 1 and 2: epidemiology, biology, and treatment. *Biological Psychiatry*.

[8] McIntyre RS, Soczynska JK, Konarski JZ (2007). Should depressive syndromes be reclassified as "metabolic syndrome type II"?. *Annals of Clinical Psychiatry*.

[9] Gans RO (2006). The metabolic syndrome, depression, and cardiovascular disease: interrelated conditions that share pathophysiologic mechanisms. *Medical Clinics of North America*.

[10] Timonen M, Laakso M, Jokelainen J, Rajala U, Meyer-Rochow VB, Keinänen-Kiukaanniemi S (2005). Insulin resistance and depression: cross sectional study. *British Medical Journal*.

[11] Skaff MM, Mullan JT, Almeida DM (2009). Daily negative mood affects fasting glucose in type 2 diabetes. *Health Psychology*.

[12] Golden SH, Lazo M, Carnethon M (2008). Examining a bidirectional association between depressive symptoms and diabetes. *Journal of the American Medical Association*.

[13] Fisher L, Mullan JT, Arean P, Glasgow RE, Hessler D, Masharani U (2010). Diabetes distress but not clinical depression or depressive symptoms is associated with glycemic control in both cross-sectional and longitudinal analyses. *Diabetes Care*.

[14] Kivimaki M, Tabak AG, Batty GD (2009). Hyperglycemia, type 2 diabetes, and depressive symptoms: the British Whitehall II study. *Diabetes Care*.

[15] Adriaanse MC, Pouwer F, Dekker JM (2008). Diabetes-related symptom distress in association with glucose metabolism and comorbidity: the Hoorn Study. *Diabetes Care*.

[16] Gale CR, Kivimaki M, Lawlor DA, Carroll D, Phillips AC, Batty GD (2010). Fasting glucose, diagnosis of type 2 diabetes, and depression: the Vietnam experience study. *Biological Psychiatry*.

[17] Kahn LS, Fox CH, McIntyre RS, Tumiel-Berhalter L, Berdine DE, Lyle H (2008). Assessing the prevalence of depression among individuals with diabetes in a medicaid managed-care program. *International Journal of Psychiatry in Medicine*.

[18] Kahn LS, Fox CH, Berdine DE, Krause-Kelly J, Raghu V (2007). A physician self-assessment tool for quality improvement. *Journal for Healthcare Quality*.

[19] Kroenke K, Spitzer RL, Williams JB (2001). The PHQ-9: validity of a brief depression severity measure. *Journal of General Internal Medicine*.

[20] Adriaanse MC, Dekker JM, Heine RJ (2008). Symptoms of depression in people with impaired glucose metabolism or type 2 diabetes mellitus: the Hoorn Study. *Diabetic Medicine*.

[21] Hennings JM, Ising M, Grautoff S, Himmerich H, Pollmächer T, Schaaf L (2010). Glucose tolerance in depressed inpatients, under treatment with mirtazapine and in healthy controls. *Experimental and Clinical Endocrinology and Diabetes*.

[22] Okamura F, Tashiro A, Utumi A (2000). Insulin resistance in patients with depression and its changes during the clinical course of depression: minimal model analysis. *Metabolism*.

[23] Lustman PJ, Griffith LS, Freedland KE, Kissel SS, Clouse RE (1998). Cognitive behavior therapy for depression in type 2 diabetes mellitus. A randomized, controlled trial. *Annals of Internal Medicine*.

